# Resection rates and intention-to-treat outcomes in borderline and locally advanced pancreatic cancer: real-world data from a population-based, prospective cohort study (NORPACT-2)

**DOI:** 10.1093/bjsopen/zrad137

**Published:** 2023-12-29

**Authors:** Ingvild Farnes, Dyre Kleive, Caroline S Verbeke, Lars Aabakken, Aart Issa-Epe, Milada Cvancarova Småstuen, Bjarte V Fosby, Svein Dueland, Pål-Dag Line, Knut J Labori

**Affiliations:** Department of Hepato-Pancreato-Biliary Surgery, Oslo University Hospital, Rikshospitalet, Oslo, Norway; Institute of Clinical Medicine, University of Oslo, Oslo, Norway; Department of Hepato-Pancreato-Biliary Surgery, Oslo University Hospital, Rikshospitalet, Oslo, Norway; Institute of Clinical Medicine, University of Oslo, Oslo, Norway; Department of Pathology, Oslo University Hospital, Rikshospitalet, Oslo, Norway; Institute of Clinical Medicine, University of Oslo, Oslo, Norway; Section of Gastroenterology, Department of Transplantation Medicine, Oslo University Hospital, Oslo, Norway; Department of Radiology, Oslo University Hospital, Oslo, Norway; Department of Health Science and Biostatistics, Oslo Metropolitan University, Oslo, Norway; Department of Transplantation Medicine, Oslo University Hospital, Oslo, Norway; Department of Oncology, Oslo University Hospital, Oslo, Norway; Institute of Clinical Medicine, University of Oslo, Oslo, Norway; Department of Transplantation Medicine, Oslo University Hospital, Oslo, Norway; Department of Hepato-Pancreato-Biliary Surgery, Oslo University Hospital, Rikshospitalet, Oslo, Norway; Institute of Clinical Medicine, University of Oslo, Oslo, Norway

## Abstract

**Background:**

Systemic chemotherapy is the initial treatment strategy for borderline resectable and locally advanced pancreatic cancer to facilitate curative resection. The aim of this study was to investigate the resection rates and overall survival in patients with borderline resectable pancreatic cancer and locally advanced pancreatic cancer.

**Methods:**

Consecutive patients with borderline resectable pancreatic cancer/locally advanced pancreatic cancer discussed by Oslo University Hospital multidisciplinary team between 2018 and 2020, serving a population of 3.1 million within a geographically defined area in south-eastern Norway, were included in this prospective Norwegian Pancreatic Cancer Trial-2 study, according to intention-to-treat principles. The total number of patients with pancreatic cancer was sought from the Cancer Registry of Norway.

**Results:**

A total of 1178 patients were diagnosed with pancreatic cancer, of whom 618 were referred to Oslo University Hospital. After multidisciplinary team evaluation, 230 patients were considered to have borderline resectable pancreatic cancer/locally advanced pancreatic cancer. The final study group consisted of 188 patients (borderline resectable pancreatic cancer *n* = 96, locally advanced pancreatic cancer *n* = 92) who were fit to receive primary chemotherapy. Resection rates were 46.9% (45 of 96) for borderline resectable pancreatic cancer and 13% (12 of 92) for locally advanced pancreatic cancer (*P* <0.001). Median overall survival was 14.6 months (borderline resectable pancreatic cancer 16.4 months; locally advanced pancreatic cancer 13.7 months, (*P* = 0.2)). Adjusted for immortal time bias, median overall survival for patients undergoing resection *versus* only chemotherapy was 24.4 months *versus* 10.1 months (*P* <0.001) for borderline resectable pancreatic cancer and 28.4 months *versus* 12.6 months for locally advanced pancreatic cancer (*P* = 0.001).

**Conclusion:**

Resection rates and survival in patients with borderline resectable pancreatic cancer and locally advanced pancreatic cancer treated at a high-volume centre in a universal healthcare system compare well with those treated at international expert centres.

Registration number: NCT04423731 (http://www.clinicaltrials.gov)

## Teaser

In this population-based, unselected, prospective cohort study, the resection rates and survival in patients with borderline resectable and locally advanced pancreatic cancer compared well with those reported by international expert centres. Patient care pathways must ensure that all borderline resectable pancreatic cancer/locally advanced pancreatic cancer patients receive appropriate evaluation with a view to surgery.

## Introduction

Pancreatic cancer is expected to be the second leading cause of cancer-related death by 2030^1^. At the time of diagnosis, more than 50% of patients present with metastatic disease. The best outcomes for patients with non-metastatic pancreatic cancer are observed in those who undergo surgical resection. However, only about 15% of patients have resectable disease at the time of diagnosis, whereas approximately 35% present with borderline resectable or locally advanced disease^2,3^. In recent years, advanced surgical resection and vascular reconstruction techniques have been developed for curative-intent surgery in borderline resectable (BRPC) and locally advanced (LAPC) pancreatic cancer, and multimodal treatment concepts have enhanced the options for surgery^4^. Practice guidelines now recognize the administration of neoadjuvant therapy as the preferred strategy for patients with BRPC^5–8^. Outcomes following folinic acid, 5-fluorouracil, irinotecan, and oxaliplatin (FOLFIRINOX) or gemcitabine/nab-paclitaxel (GnP) treatment in patients with LAPC are also promising^9–11^. In a proportion of patients with LAPC, primary chemotherapy may result in downstaging to (borderline) resectable disease and offers the possibility of surgical resection if venous and/or arterial en-bloc resection and reconstruction can be achieved^9–11,12–14^. Although neoadjuvant chemoradiotherapy is associated with increased rates of negative resection margin, a survival benefit of neoadjuvant chemoradiotherapy over neoadjuvant chemotherapy alone has not been shown^15^.

Several high-volume expert centres have reported high resection rates and favourable outcomes following primary chemotherapy in patients with BRPC and LAPC^12–14,16^. However, in these studies, intention-to-treat survival analysis is seldom performed, and there is a risk of selection bias inherent to referral for such operations to these expert centres^9,17,18^. There is a need for studies presenting the true denominator based on which resection rates and outcomes are calculated^17^. The aim of the present study was to investigate the resection and survival rates of BRPC and LAPC in a prospective, consecutive and population-based cohort of patients treated in a universal healthcare system after the routine introduction of modern chemotherapy regimens.

## Methods

### Study population, study design and definitions

Norway has a universal public healthcare system organized into four independent regional health authorities. Oslo University Hospital (OUH) is the largest centre and covers a population of 3.1 million people (of a total population of 5.4 million) within a geographically defined area (South-Eastern Norway Regional Health Authority (S-ENRHA))^19^. Venous resection is the institutional standard of practice, and selected patients proceed to arterial resection, as previously described^20–22^.

Consecutive patients with BRPC and LAPC referred from the S-ENRHA to the multidisciplinary team (MDT) at OUH between 1 January, 2018 and 31 December, 2020 were prospectively included. Patients were classified as having BRPC or LAPC based on the National Comprehensive Cancer Network (NCCN) criteria, version 2, 2017^8^. Patients were offered participation in this prospective, observational study and 230 patients were included following written informed consent. No patients declined inclusion. The study protocol was approved by the Regional Ethical Committee (REC Nord 2017/1382, Norwegian Pancreatic Cancer Trial-2 (NORPACT-2)) in August 2017. Clinical data were prospectively recorded. REC waived written informed consent for patients who were not alive at the date of last follow-up in cases in which written informed consent was not obtained because the patient did not visit OUH for the diagnostic work-up. A crosscheck with the Cancer Registry of Norway was performed to ensure that the patient cohort was representative of the S-ENRHA population. Patients who had been referred from the other three regional health authorities to OUH for a second opinion and possible resection were excluded. The study was conducted in accordance with the STROBE guidelines^23^.

**Fig. 1:**
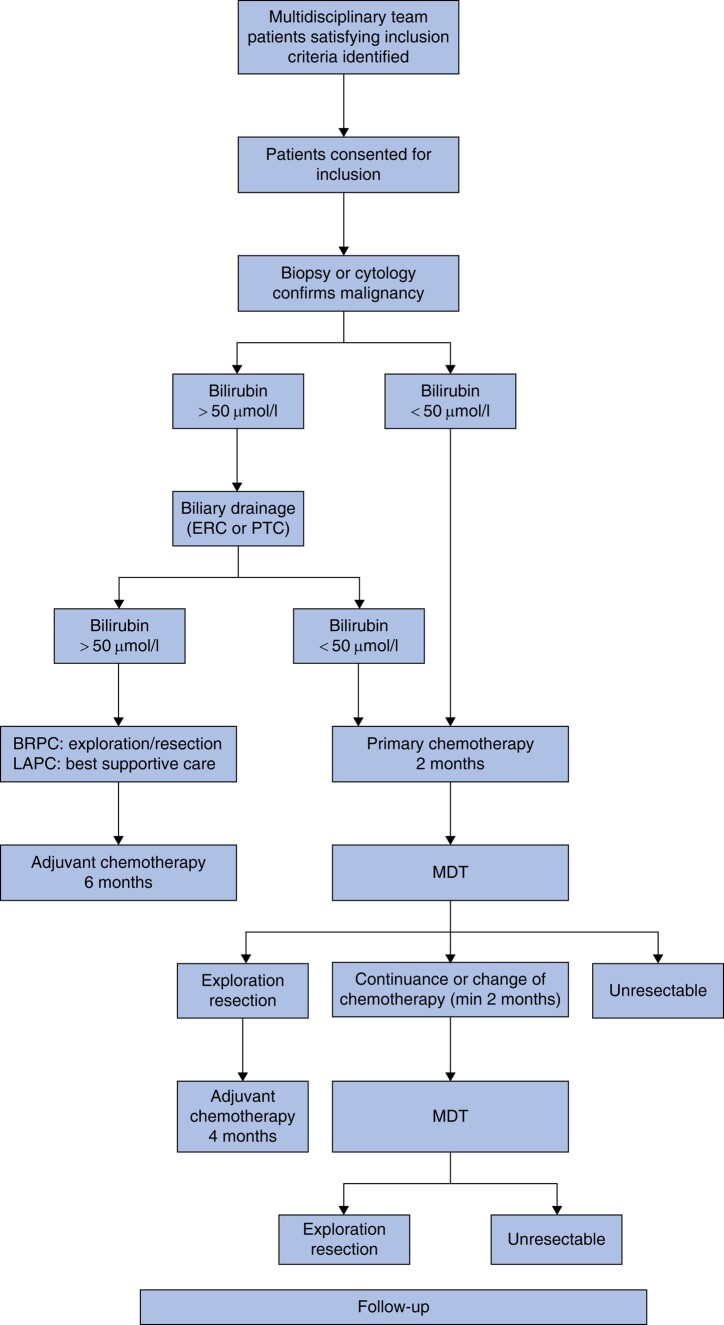
Study design ERC, endoscopic retrograde cholangiography; PTC, percutaneous transhepatic cholangiogram; BRPC, borderline resectable pancreatic cancer; LAPC, locally advanced pancreatic cancer; MDT, multidisciplinary team.

The diagnostic work-up, treatment sequence and surgical and medical interventions were performed in accordance with the national guidelines (*Fig. 1*)^5^. In Norway, neoadjuvant or induction chemotherapy is recommended for all BRPC/LAPC patients. Before study inclusion, distant metastases were ruled out using contrast-enhanced dual-phase multislice computed tomography (CT) scans of the abdomen and chest. Fine-needle aspiration cytology/biopsy (FNA/FNB) guided by endoscopic ultrasound was required to confirm pancreatic cancer prior to chemotherapy. An experienced abdominal radiologist evaluated vascular involvement of the coeliac trunk, superior mesenteric artery, hepatic artery and portomesenteric veins. The NCCN classification was performed by an experienced team of abdominal radiologists and pancreatic surgeons, and reviewed by two of the authors (I.F., K.J.L). Changes in tumour during chemotherapy were described using the modified Response Evaluation Criteria in Solid Tumours (RECIST) (version 1.1)^24^. In cases of LAPC, venous and/or arterial involvement had to be evaluated and considered potentially resectable by two hepato-pancreato-biliary surgeons and one abdominal transplant surgeon. Performance status (Eastern Cooperative Oncology Group (ECOG)) and the Charlson co-morbidity index (CCI) (https://www.mdcalc.com/charlson-comorbidity-index-cci) were recorded at the time of diagnosis. The points given in the CCI for the diagnosis of a solid tumour and age were excluded from the final points given. Carbohydrate antigen (CA) 19-9 levels were measured at baseline and at response evaluations. Levels associated with total bilirubin >1.5 mg/dl and CA 19-9 levels <5 U/ml both before and after treatment (non-secretors) were excluded from the analysis.

### Chemotherapy and surgery

Chemotherapy was administered according to different regimens, preferably FOLFIRINOX, in fit patients^5^. The chemotherapy regimen was decided by the treating oncologist at the patient`s local hospital. (m)FOLFIRINOX, Fluorouracil/Leucovorin/Oxaliplatin (FLOX) or Fluorouracil/Leucovorin (FLV) were administered every 2 weeks (one cycle). Gemcitabine or GnP were administered on days 1, 8 and 15 every 3 weeks (one cycle). Dose reduction or dose delays were introduced according to the local guidelines at each centre. Grade 3–5 side effects of chemotherapy were registered (Common Terminology Criteria for Adverse Events version 5; http://ctep.cancer.gov/forms/CTCAEv5.pdf). For BRPC, exploration was recommended after 2 months of chemotherapy in cases of stable disease or response and for LAPC after 4 months in case of response. Restaging was performed using a CT scan of the chest and abdomen, and CA19-9 was measured after completion of four (m)FOLFIRINOX, FLOX, or FLV cycles, or two cycles of gemcitabine or GnP.

Within 4 weeks of the last neoadjuvant infusion, surgical resection was performed as a standard or pylorus-preserving pancreatoduodenectomy (PD), distal pancreatectomy (DP) with splenectomy or total pancreatectomy (TP) with splenectomy. PD reconstruction was performed by retrocolic end-to-side pancreatojejunostomy and an end-to-side hepaticojejunostomy. In addition, an end-to-side duodenojejunostomy or gastrojejunostomy was performed. Venous and arterial resections and reconstruction were undertaken in collaboration with an abdominal transplant surgeon.

Patients who underwent surgical resection received adjuvant chemotherapy, preferably mFOLFIRINOX. However, gemcitabine/capecitabine, gemcitabine or FLV were administered at the discretion of the treating oncologist^5^. Adjuvant chemotherapy was initiated within 12 weeks of the resection. The total duration of chemotherapy in the resected patients was 6 months. Follow-up in resected patients included CA19-9 and CT scans of the chest and abdomen at 6 and 12 months after surgery, and annually thereafter until disease recurrence or, in patients without relapse, until 5 years after surgery. Overall survival (OS) was recorded by the Norwegian Population Registry. Survival was defined as the time from the date of CT diagnosis to the date of death from any cause or the end of follow-up through to 15 September, 2022.

### Statistical analysis

Statistical analyses were performed using SPSS version 27 and STATA version 17. Continuous variables are described as medians with interquartile ranges and compared using the Mann–Whitney test or Kruskal–Wallis test. Categorical variables are expressed as counts with % and compared using the χ^2^ or Fisher’s exact test (for small numbers). OS was measured from the time of diagnosis until death or the last follow-up. Crude differences between patient groups were assessed using the Kaplan–Meier method and the log-rank test. Survival analysis of resected patients was subject to immortal time bias since patients undergoing resection had to survive for a sufficient interval of time from diagnosis to receive surgery, artificially inflating the survival benefit in this group. An extended Cox model was used to adjust for immortal time bias. In this model, all patient data were used, and a time-varying covariate (here, being resected or not) in the model tracked whether the classifying event (here, death) occurred during the estimation process^25^. All patients were initially classified into the chemotherapy-only group and as not undergoing resection. Patients who underwent resection were initially analysed as not resected and switched to the resection group at the date of resection, where they remained until relapse or death. The extended Cox model offers the advantage of using all study follow-up data because the analysis starts at the time of enrolment for all included patients, regardless of their future resection status. Thus, in contrast to the conditional landmark approach, a higher level of statistical power is maintained. Univariate and multivariate Cox proportional hazards regression models were used to assess possible independent prognostic factors for OS. Univariate and multivariate logistic regression models were used to identify possible independent predictors of surgical resection. Variables that reached a *P* value <0.2 in univariate analyses were entered into multivariate models. All analyses were considered exploratory; therefore, no correction for multiple testing was carried out and all tests were two-sided. Statistical significance was set at *P* <0.05.

## Results

### Overall cohort characteristics

**Fig. 2:**
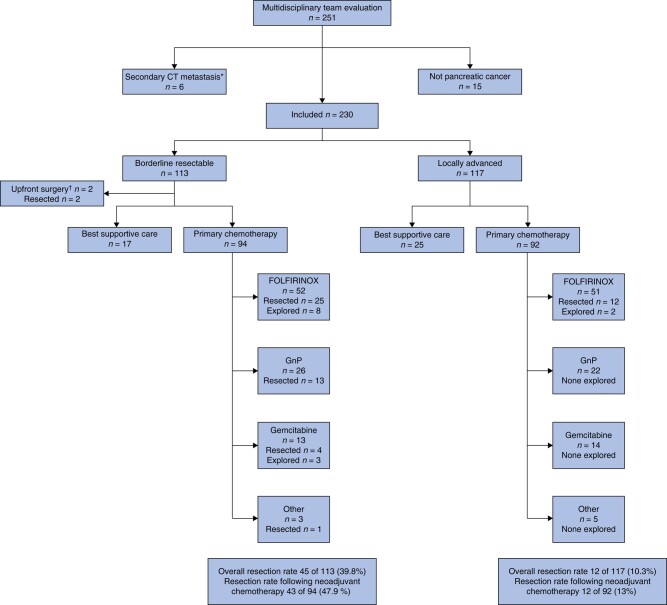
Flow diagram of the study stratified by resectability status according to the National Comprehensive Cancer Network classification (borderline resectable or locally advanced), primary treatment (best supportive care, primary chemotherapy or upfront surgery) and first-line chemotherapy regimen (FOLFIRINOX, gemcitabine/nab-paclitaxel, gemcitabine, other) Outcome of surgery is classified as resected or explored only. *Secondary CT performed in six patients before initiation of planned neoadjuvant chemotherapy showed distant metastasis, and the patients received palliative chemotherapy. ^†^Two patients with borderline resectable pancreatic cancer underwent upfront surgery followed by adjuvant chemotherapy. One patient with borderline resectable pancreatic cancer received fluorouracil/leucovorin/oxaliplatin and underwent subsequent resection. CT, computed tomography; FOLFIRINOX, fluorouracil, leucovorin, oxaliplatin and irinotecan; GnP, gemcitabine nab-paclitaxel.

Of 230 patients, referred to and discussed at the MDT meeting at OUS, 113 (49.1%) had BRPC and 117 (50.9%) LAPC (*Fig. 2*). Forty-two patients (18.2%) received best supportive care only. These patients, presented in *Table S1*, were excluded from further analysis. Of the remaining 188 patients, 186 patients (98.9%) received primary chemotherapy, while 2 BRPC patients (1.1%) underwent upfront surgery followed by adjuvant chemotherapy (*Fig. 2*). Baseline characteristics of patients fit to receive chemotherapy are presented in *Table 1*, stratified by resectability status. None of the patients were lost to follow-up. There were no differences in sex, BMI, ECOG status, CCI, CA 19-9 and assigned chemotherapy between the groups. However, age, tumour diameter, tumour location and rate of biliary drainage differed between the groups.

**Table 1:** Baseline and treatment characteristics for patients receiving chemotherapy stratified by resectability status according to the National Comprehensive Cancer Network classification

	Overall* (*n* = 188)	Borderline resectable* (*n* = 96)	Locally advanced (*n* = 92)	*P*
Age (years)	69.5 (63–74)	72 (64.5–74.5)	67 (60.5–72)	0.009
**Sex**				
Male	98	47	51	0.374
Female	90	49	41	0.374
BMI (kg/m^2^)	23.7 (21–26.5)	23.5 (21.3–25.9)	23.9 (21–26.8)	0.486
**Performance status (ECOG)**				
0	110 (58.5)	52 (54.2)	58 (63.1)	0.405
1	64 3 (34.1)	37 (38.5)	27 (29.3)	
>1	14 (7.4)	7 (7.3)	7 (7.6)	
**Charlson co-morbidity index**				
0	95 (50.5)	48 (50)	47 (51.1)	0.794
1	61 (32.4)	33 (34.4)	28 (30.4)	
>1	32 (17.1)	15 (15.6)	17 (18.5)	
Biliary drainage	100	63 (65.6)	37 (40.2)	<0.001
CA19-9 (kU/l)	343 (75–1001)	323 (72–953)	345 (76–1038)	0.734
**Tumour location**				
Head/uncinate process	153 (81.4)	87 (90.6)	66 (71.7)	<0.001
Body/tail	35 (18.6)	9 (0.4)	26 (28.3)	
Tumour size prechemotherapy (mm)	35.5 (29–44.8)	30 (25.5–36.5)	40.5 (35–50)	<0.001
Time from diagnosis to initiation of chemotherapy, days	44.5 (32–57)	45 (32–60)	44.5 (32–56)	0.863
**Chemotherapy regimen**				
FOLFIRINOX	103 (54.8)	52 (54.2)	51 (55.4)	0.592
Gemcitabine/nab-paclitaxel	48 (25.5)	26 (27.1)	22 (23.8)	
Gemcitabine	27 (14.4)	13 (13.5)	14 (15.2)	
Other regimen†	8 (4.3)	3 (3.1)	5 (5.4)	
Upfront surgery	2 (1.1)	2 (2.1)	0 (0)	
**Number of cycles**				
FOLFIRINOX	4 (3–5)	4 (3–4)	4 (3.5–7)	0.052
Gemcitabine/nab-paclitaxel	2 (2–3.8)	2 (1–2)	2 (2–4)	0.148
Gemcitabine	2 (1–3)	2 (1–3)	2 (1–3)	0.830
Other regimen†	NA	NA	NA	NA
Chemotherapeutic switch	30 (16)	12 (12.5)	18 (19.6)	0.186
CTCAE grade 3–5 adverse events	100 (53.2)	49 (51)	51 (55.4)	0.546
CA19-9 postchemotherapy (kU/l)‡	210 (56–617)	166 (56–541)	222 (63–641)	0.573
**CA19-9 dynamics‡**				
Normalization	14 (7.5)	6 (6.4)	8 (8.7)	0.550
>50% decrease	58 (31.2)	31 (33)	27 (29.3)	0.593
<50% decrease	35 (18.8)	19 (20.2)	16 (17.4)	0.623
Increase	47 (25.3)	22 (23.4)	25 (27.1)	0.554
No change (<37 kU/l)	14 (7.5)	7 (7.4)	7 (7.6)	0.967
Tumour size after chemotherapy (mm)	33 (25–45)	27 (21–33)	40 (30–53)	<0.001
**Response Evaluation Criteria In Solid Tumours at restaging§**				
Complete/partial response	18 (9.9)	10 (10.9)	8 (8.9)	0.785
Stable disease	121 (67.6)	62 (67.4)	59 (65.5)	
Progressive disease	43 (23.6)	20 (21.7)	23 (25.6)	
Time from diagnosis to definitive decision of resectability by the MDT	112 (89–158)	106 (84–129)	132 (95–175)	0.005
**Definitive decisions by the MDT§**				
Surgical exploration	70 (37.2)	56 (58.3)	14 (15.2)	<0.001
Palliative treatment/best supportive care for metastases	28 (14.9)	14 (14.6)	14 (15.2)	
Palliative treatment/best supportive care for unresectable disease	78 (41.5)	24 (25)	54 (58.7)	
Unknown status	12 (6.4)	2 (2.1)	10 (10.9)	
Resection rate	57 (30.3)	45 (46.9)	12 (13)	<0.001
**Type of procedures**				
Pancreatoduodenectomy	49 (86)	42 (93.3)	7 (58.4)	0.002
Distal pancreatectomy	3 (5.3)	2 (4.5)	1 (8.3)	
Total pancreatectomy	5 (8.7)	1 (2.2)	4 (33.3)	
Concomitant vascular resection	34 (59.6)	26 (57.8)	8 (66.7)	0.577
Postoperative complications ≥ Clavien grade III	14 (24.6)	8 (17.8)	6 (50)	0.021
Adjuvant chemotherapy	35 (61.4)	32 (71.1)	3 (25)	0.006

Values are *n* (%) or median (interquartile range) unless otherwise stated. *Two patients undergoing upfront surgery included only in analysis of MDT decisions and outcome of surgery. †fluorouracil/leucovorin/oxaliplatin, *n* = 6; nab-paclitaxel, *n* = 1; regional chemotherapy, *n* = 1. ‡CA19-9 at baseline: missing *n* = 11, hyperbilirubinaemia *n* = 2, non-secretor (<5 kU/l) *n* = 12. §Two borderline resectable and two locally advanced patients did not undergo the planned first restaging computer tomography (*Figs S1*, *S2*), and eight locally advanced patients did not undergo the planned second restaging computer tomography (*Fig. S4*). CA19-9, carbohydrate antigen 19-9; CTCAE, Common Terminology Criteria for Adverse Events; ECOG, Eastern Cooperative Oncology Group; FOLFIRINOX, 5-fluorouracil with leucovorin, irinotecan and oxaliplatin; MDT, multidisciplinary team; NA, not applicable. BMI, missing data *n* = 3.

The primary chemotherapy regimens were FOLFIRINOX in 103 patients (55.4%), GnP in 48 (25.8%), gemcitabine in 27 (14.5%) and other regimens in 8 (4.3%) (*Table 1*, *Fig. 2*). Patients receiving FOLFIRINOX were significantly younger and had a higher BMI and better performance status (*Table S2*). Information regarding the MDT decisions and response evaluations, and causes of not undergoing exploration/resection are detailed in *Table 1* and *Figs S1*, *S2*. During primary chemotherapy, 30 patients (16.1%) underwent chemotherapeutic switch before the final MDT decision, in the majority of whom FOLFIRINOX was the first-line regimen. Details of grade 3–5 toxicity occurring during primary chemotherapy are presented in *Table S3*. Overall, 100 patients (53.8%) experienced grade 3–5 adverse events. Fatal adverse events occurred in two patients.

The median time from diagnosis to a definitive decision by the MDT regarding resectability was significantly lower in the BRPC group than in the LAPC group (106 and 132 days respectively) (*P* = 0.005). RECIST response was available for 182 patients: 18 (9.9%) had partial/complete response (partial *n* = 16, complete *n* = 2), 121 (66.5%) stable disease and 43 (23.6%) disease progression (*Table 1*). CA19-9 values at both baseline and time of response evaluation were available from 157 (84.4%) patients. CA19-9 reduction of >50% was observed in 58 patients (31.2%) and CA19-9 normalization in 14 patients (7.5%) (*Table 1*).

### Resection rates and predictive factors of eventual resection

Of 113 patients with BRPC, 56 were explored and 45 (39.8%) patients underwent resection (*Fig. 2*). In patients receiving chemotherapy, the resection rate for BRPC was 45.7% (43 of 94) (*Fig. 2*). Of 117 patients with LAPC, 14 underwent exploration and 12 (10.3%) patients underwent resection (*Fig. 2*). In patients receiving chemotherapy, the resection rate for LAPC was 13% (12 of 92) (*Fig. 2*). Reasons for not undergoing exploration are presented in *Figs S1*, *S2*. In the multivariate analysis, LAPC (OR 0.20, *P* <0.001), CA19-9 >500 (OR 0.44, *P* = 0.023) and treatment with gemcitabine (OR 0.09, *P* = 0.023) were negative predictors of eventual resection (*Table 2*). A cross-check with the Cancer Registry of Norway revealed a total of 1178 cases of all stages of pancreatic cancer in the S-ENRHA population during the study period, of which 618 (52.5%) were referred to OUH and 249 (resectable, BRPC and LAPC) underwent resection (overall population-based pancreatic cancer resection rate 21.1%) (*Fig. S3*).

**Table 2:** Univariate and multivariate analysis of variables associated with surgical resection (logistic regression) and overall survival (Cox regression) in patients fit to receive chemotherapy using baseline factors for all patients

Baseline variable	Number	Resection				Survival			
		Univariate		Multivariate		Univariate		Multivariate	
		Odds ratio	*P*	Odds ratio	*P*	Hazard ratio	*P*	Hazard ratio	*P*
**Sex**									
Female	90	1 (Ref)	NA			1 (Ref)	NA		
Male	98	1.40 (0.75,2.61)	0.297			0.80 (0.58,1.10)	0.163		
**Age (years)**									
≤75	148	1 (Ref)	NA			1 (Ref)	NA		
>75	40	0.60 (0.27,1.37)	0.228			1.37 (0.94,2.00)	0.102		
**Performance status (ECOG)**									
0	110	1 (Ref)	NA			1 (Ref)	NA	1 (Ref)	NA
1	64	1.02 (0.52,1.98)	0.963			0.96 (0.68,1.35)	0.807	0.86 (0.58,1.27)	0.436
>1	14	0.61 (0.16,2.33)	0.469			2.02 (1.15,3.57)	0.015	1.81 (0.91,3.61)	0.091
BMI (kg/m^2^)*									
18.5–30	150	1 (Ref)	NA			1 (Ref)	NA		
<18.5	17	0.94 (0.31,2.83)	0.915			0.97 (0.55,1.72)	0.914		
≥30	18	1.13 (0.40,3.20)	0.817			0.78 (0.45,1.36)	0.390		
**Charlson co-morbidity index**									
0	95	1 (Ref)	NA			1 (Ref)	NA		
1	61	1.03 (0.51,2.06)	0.935			0.86 (0.60,1.22)	0.388		
>1	32	0.89 (0.37,2.16)	0.798			1.06 (0.68,1.67)	0.793		
**Tumour size (mm)**									
0–20	15	1 (Ref)	NA			1 (Ref)	NA		
21–40	115	1.77 (0.53,5.89)	0.354			0.67 (0.37,1.19)	0.170		
>40	58	0.44 (0.11,1.73)	0.239			0.76 (0.41,1.40)	0.374		
**Tumour location**									
Caput	153	1 (Ref)	NA			1 (Ref)	NA		
Corpus/cauda	35	0.52 (0.21,1.26)	0.146			1.07 (0.72,1.61)	0.732		
**Anatomic tumour classification**									
Borderline resectable	96	1 (Ref)	NA	1 (Ref)	NA	1 (Ref)	NA		
Locally advanced	92	0.17 (0.08,0.35)	<0.001	0.20 (0.09,0.46)	<0.001	1.22 (0.89,1.67)	0.224		
**CA19-9 (kU/l)†**									
5–500	96	1 (Ref)	NA	1 (Ref)	NA	1 (Ref)	NA	1 (Ref)	NA
>500	67	0.44 (0.21,0.92)	0.028	0.44 (0.19,0.98)	0.045	1.83 (1.28,2.60)	<0.001	1.65 (1.12,2.41)	0.010
**Primary chemotherapy regimen‡**									
FOLFIRINOX	103	1 (Ref)	NA	1 (Ref)	NA	1 (Ref)	NA	1 (Ref)	NA
Gemcitabine/nab-paclitaxel	48	0.66 (0.31,1.41)	0.284	0.68 (0.28,1.62)	0.379	1.50 (1.03,2.18)	0.034	1.43 (0.95,2.16)	0.084
Gemcitabine	27	0.31 (0.10,0.97)	0.043	0.09 (0.01,0.71)	0.023	1.71 (1.08,2.68)	0.021	1.78 (1.02,3.11)	0.042

Values in parentheses are 95% confidence intervals. ‡Eight patients received other chemotherapy regimens, and two patients underwent upfront surgery followed by adjuvant chemotherapy. CA19-9, carbohydrate antigen 19-9; ECOG, Eastern Cooperative Oncology Group; FOLFIRINOX, 5-fluorouracil with leucovorin, irinotecan and oxaliplatin; NA, not applicable. *BMI, missing data *n* = 3; †CA19-9 at baseline, missing data *n* = 11, hyperbilirubinaemia *n* = 2, non-secretor *n* = 12.

### Survival and prognostic factors

**Fig. 3:**
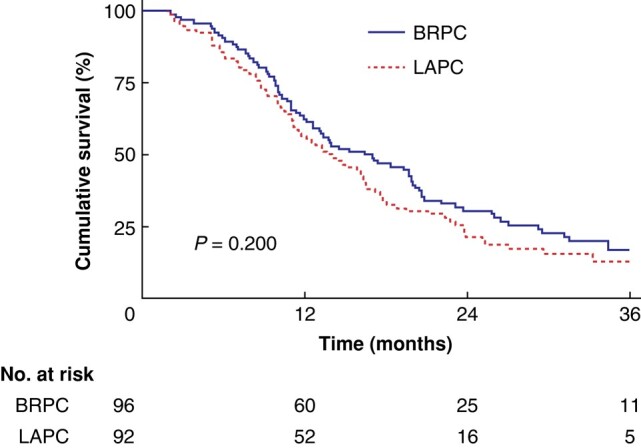
Overall survival for the 188 patients, regardless of being resected or not, receiving chemotherapy stratified by resectability status (borderline resectable *versus* locally advanced) according to the National Comprehensive Cancer Network classification BRPC, borderline resectable pancreatic cancer; LAPC, locally advanced pancreatic cancer.

**Fig. 4:**
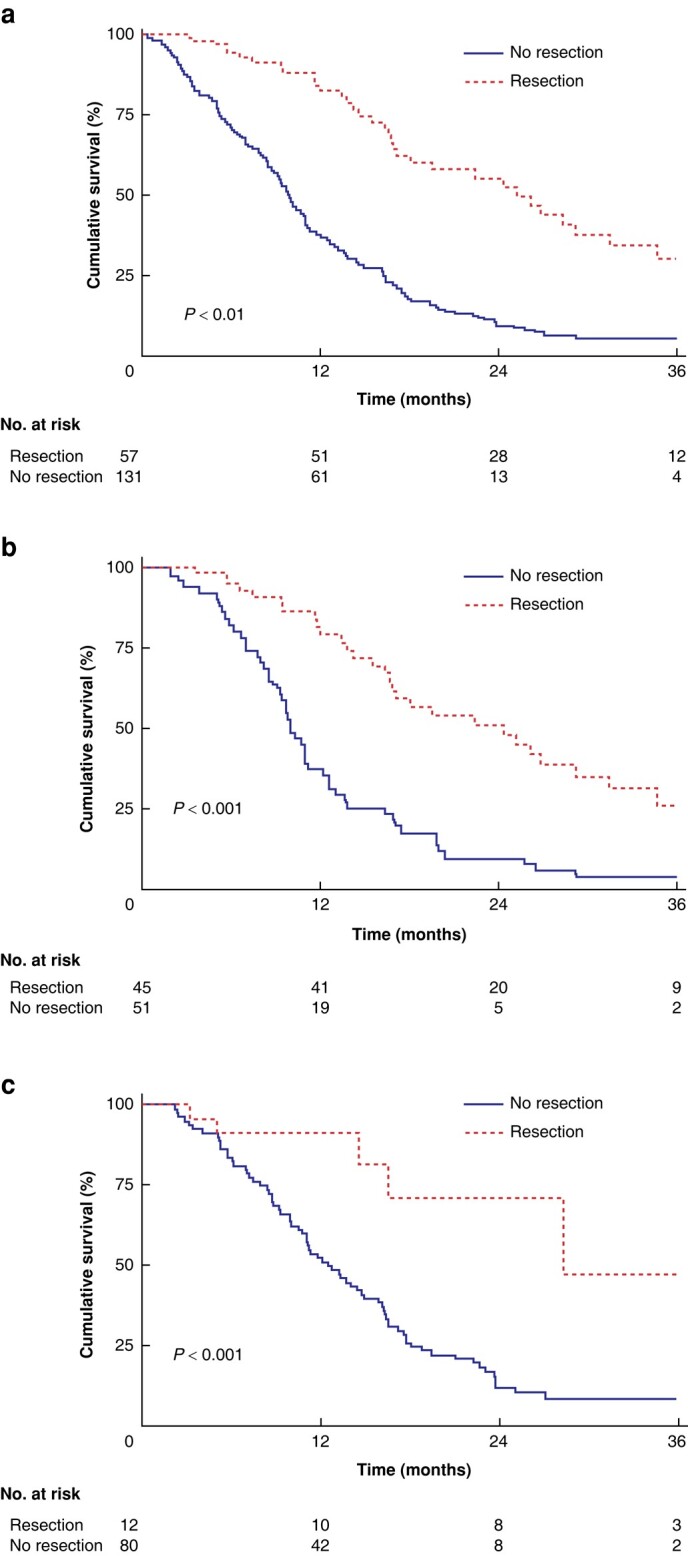
Overall survival for the study cohort **a** Overall survival adjusted for immortal time bias for 188 patients with borderline resectable and locally advanced pancreatic cancer receiving chemotherapy stratified by treatment (resection *versus* no resection). **b** Overall survival adjusted for immortal time bias for 96 patients with borderline resectable pancreatic cancer receiving chemotherapy stratified by treatment (resected *versus* non-resected). **c** Overall survival adjusted for immortal time bias for 92 patients with locally advanced pancreatic cancer receiving chemotherapy stratified by treatment (resected *versus* non-resected).

After a median follow-up of 14.7 months (95% c.i. 1.9 to 36), 156 of 188 patients (83%) had died. OS of all patients was 14.6 months (95% c.i. 12.7 to 17.2). For BRPC and LAPC patients, OS was 16.4 (95% c.i. 12.6 to 19.9) and 13.7 (95% c.i. 11.2 to 16.6) months respectively (*P* = 0.2) (*Fig. 3*). OS from time of diagnosis in resected patients was 29.5 months (95% c.i. 23.1 to 34.4), 27 months (95% c.i. 20.7 to 34.4) for BRPC and 33.2 months (95% c.i. 11.5 to not estimated) for LAPC. OS from time of diagnosis in non-resected patients was 11.2 months (95% c.i. 10 to 13.1), 10 months (95% c.i. 8.6 to 12.2) for BRPC and 12.6 months (95% c.i. 10.5 to 15.9) for LAPC. After accounting for immortal time bias in the adjusted analysis, OS was 25.3 months (95% c.i. 17.1 to 31.5) in resected patients *versus* 11.2 months (95% c.i. 10 to 13.1) in non-resected patients (*P* < 0.001) (*Fig. 4a*) (BRPC: 24.4 months (95% c.i. 16.7 to 31.5) *versus* 10.1 months (95% c.i. 8.6 to 12.2), (*P* < 0.001); LAPC: 28.4 months (95% c.i. 14.6 to not estimated) *versus* 12.6 (95% c.i. 10.5 to 15.9), (*P* = 0.001); (*Fig. 4b*, *c*). In the multivariate analysis, CA19-9 >500 (HR 1.65, *P* = 0.010) and treatment with gemcitabine (HR 1.78, *P* = 0.042) were independent, negative prognostic factors (*Table 2*). The cross-check with the Cancer Registry of Norway showed that patients not referred to OUH were older and had poor survival compared with patients evaluated at OUH (*Fig. S3*).

## Discussion

This population-based study shows that resection rates and survival following primary chemotherapy in patients with BRPC and LAPC treated in a universal healthcare system compare well with those treated in international expert centres. Surgical resection was associated with improved survival in both groups. However, although resection rates varied significantly between patients with BRPC and those with LAPC, they did not constitute distinct prognostic groups.

A recent meta-analysis showed resection rates of 60.6% for BRPC and 22.2% for LAPC following primary chemo(radio)therapy, and surgical resection was associated with improved survival compared with chemo(radio)therapy only (BRPC, 32.3 *versus* 13.9 months; LAPC, 30.0 *versus* 14.6 months)^9^. Of note, the prediction interval, which incorporates both within- and between-study variances in order to present the expected range of resection rates in similar studies, showed a confidence interval for the resection rate in LAPC of 4.3 to 64.1%^9^. Recent series from high-volume expert centres report on resection rates in LAPC between 18% and 50.8%^12,14,26,27^. These centres may overestimate resection rates and survival due to selection biases inherent to referral for such operations to these centres, the lack of an intention-to-treat approach and the lack of a population-based study design. Moreover, the definitions of BRPC and LAPC differ between the studies. A better insight into the resection rates and outcomes in BRPC and LAPC was recently provided by two studies that contextualized the outcomes of patients with BRPC and LAPC relative to a meaningful population-at-risk^28,29^. However, the Italian study (resection rate 24.1% in BRPC and 9% in LAPC) did not have a population-based design, and the Dutch study (13% resection rate in LAPC) did not use the NCCN definition of LAPC^28,29^. This population-based study used the widely accepted NCCN criteria to define BRPC and LAPC. Compared with data based on selected patient cohorts from international expert centres or clinical trials with strict inclusion criteria, the present study provides more realistic results regarding resection rates, treatment outcomes and survival.

After adjusting for immortal time bias, OS was favourable in patients undergoing resection. As the best outcomes for patients with BRPC or LAPC are seen in those who undergo successful surgical resection, the decision to resect or not is key. Accordingly, patient care pathways must ensure that all patients receive appropriate evaluations with the prospect of surgical resectability. Based on the data from this study, a centre with a catchment area population of 3.1 million should expect to manage on average 75–80 patients with BRPC and LAPC per year, whereas resections would be appropriate for approximately 50% of BRPC (≍15 cases per year) and 10% of LAPC (≍5 cases per year). Providing neoadjuvant/primary therapy reliably and safely to patients with pancreatic cancer is not trivial, and developing the required infrastructure and support has been a long process, even in experienced centres^30^. The team must be able to perform accurate pre- and posttreatment staging, evaluate toxicity and response during primary chemo-(radio) therapy, consider chemotherapeutic switch, evaluate resectability and undertake complex vascular resections. In the Netherlands, the ongoing PREOPANC-4 trial aims to achieve a safe and patient-centred nationwide implementation of the international standards of excellence for LAPC surgery^31^. In Scandinavian countries, the centralization of care for patients with BRPC or LAPC is under debate. Pancreatic surgery has been considered one of the most sensitive procedures to the effect of centralization^32^. The Improving Outcomes Guidance document, which was published in 2001, recommended centralization of pancreatic surgery in England and Wales for populations up to 4 million. The English model seems well arranged to ensure a pancreatic cancer specialist team with experience in caring for patients with BRPC or LAPC^33^.

Surgery in BRPC and LAPC requires patients to endure a clinical pathway that includes a lengthy and rigorous course of chemotherapy, followed by complex surgery. Grade 3–5 toxicity during chemotherapy and major surgical complications in this study occurred in 53.3% and 24.6% respectively. At the time of diagnosis, an overall realistic understanding of the patient's likelihood of becoming eligible for surgical resection and overall prognosis is important. Baseline characteristics were specified before the occurrence of any outcome event and used in this study to predict resectability and prognosis. Patients with non-metastatic pancreatic cancer are a heterogeneous population and are categorized based on tumour anatomy (resectable, BRPC, LAPC), tumour biology and patient physiology. This study shows that patients with anatomically defined BRPC according to the NCCN criteria are more likely to undergo resection. However, the 4-fold higher resection rate was insufficient to affect the collective survival outcome. This finding is in line with other reports that discourage the use of the NCCN distinction between BRPC and LAPC to define different prognostic categories^26,28,34^. However, baseline CA19-9 >500, which is commonly used to define aggressive tumour biology in pancreatic cancer, was an independent predictor of worse survival^35^. A cross-check with the Cancer Registry of Norway revealed that patients with localized tumours or regional spread, who were not evaluated at the study hospital, had a median age of >80 years and poor survival. Most probably, patients with poor physiology due to old age, poor co-morbidity profile and poor performance status were not referred to the MDT for consideration of surgery. In our opinion, this study presents a representative population-based cohort of patients who are fit to receive chemotherapy and eventual surgery for BRPC or LAPC.

Strengths of this study include a population-based, non-selected cohort treated in a universal healthcare system. Consecutive patients were prospectively enrolled in this protocol-based study by means of an intention-to-treat principle. Moreover, the widely accepted NCCN criteria for defining BRPC and LAPC were used. This study has some limitations. First, the number of patients is relatively small for a comparison of the two main primary chemotherapy regimens that were used. Second, a cross-check with the Cancer Registry of Norway revealed that a proportion of the population with non-metastatic pancreatic cancer was not referred to the study hospital. However, these patients were mainly older patients treated with best supportive care who had a short survival. This bias is inevitable and most studies do not provide insights into these numbers. Finally, as this is a single-centre study, external validity may be limited. However, the catchment area of 3.1 million ensures surgical experience and a dedicated team within a high-volume setting that is essential for optimal selection, surgical treatment, adequate management of postoperative complications and international standard of care for LAPC^36^.

## Supplementary Material

zrad137_Supplementary_Data

## Data Availability

The data presented in this study are available upon reasonable request from the corresponding author. The data are not publicly available due to privacy regulations.
