# Supplementary material for: Resection rates and intention-to-treat outcomes in borderline and locally advanced pancreatic cancer: real-world data from a population-based, prospective cohort study (NORPACT-2)
Source: BJS Open. 2023 Dec 29;7(6):zrad137. doi: 10.1093/bjsopen/zrad137 (PMC10755199; doi:10.1093/bjsopen/zrad137)
Supplement: zrad137_Supplementary_Data [file zrad137_supplementary_data.docx]

**Resection rates and intention-to-treat outcomes in borderline and locally advanced pancreatic cancer - Real-world data from a population-based, prospective cohort study (NORPACT-2)**

Ingvild Farnes^1,2^ MD, Dyre Kleive^1^ MD PhD, Caroline S. Verbeke^2,3^ MD PhD, Lars Aabakken^2,4^ MD PhD, Aart Issa-Epe^5^ MD, Milada Cvancarova Småstuen^6^ MSc PhD, Bjarte V. Fosby^7^ MD PhD, Svein Dueland^8^ MD PhD, Pål-Dag Line^2,7^ MD PhD, Knut J. Labori^1,2^ MD PhD

^1^Department of Hepato-Pancreato-Biliary Surgery, Oslo University Hospital, Rikshospitalet, Oslo, Norway

^2^Institute of Clinical Medicine, University of Oslo, Oslo, Norway

^3^Department of Pathology, Oslo University Hospital, Rikshospitalet, Oslo, Norway

^4^Section of Gastroenterology, Department of Transplantation Medicine, Oslo University Hospital, Oslo, Norway

^5^Department of Radiology, Oslo University Hospital, Oslo, Norway

^6^Department of Health Science and Biostatistics, Oslo Metropolitan University, Norway

^7^Department of Transplantation Medicine, Oslo University Hospital, Oslo, Norway

^8^Department of Oncology, Oslo University Hospital, Oslo, Norway

**Corresponding author:** Ingvild Farnes, MD, Nydalen 0424, Oslo, Norway Telephone: + 47 23070000, Fax number: +47 23072526 E-mail: [infarn@ous-hf.no](mailto:infarn@ous-hf.no)

| **Supplementary Figures and Tables** |  |
| --- | --- |
| Supplementary Figure 1: Page 2  Supplementary Figure 2: Page 3  Supplementary Figure 3: Page 4  Supplementary Table 1: Page 5  Supplementary Table 2: Page 6  Supplementary Table 3: Page 7 |  |

**Supplementary Figure 1:** Flowchart showing multidisciplinary team decisions at baseline and during response evaluations for patients receiving chemotherapy for borderline resectable pancreatic cancer.

**Supplementary Figure 2:** Flowchart showing multidisciplinary team decisions at baseline and during response evaluations for patients receiving chemotherapy for locally advanced pancreatic cancer.

**Supplementary Figure 3:** All cases of pancreatic cancer (ICD-10 code C25, excluding C25.4 neuroendocrine tumors) in the South Eastern Regional Health Authority (catchment area 3.1 million) in the study period 2018-2020. In a total of 1178 cases of pancreatic cancer, 618 were referred to Oslo University Hospital, and 249 underwent resection (resection rate 21.1 %). Values are number, age (years) and overall survival (months (95 % confidence interval)). Date of last follow up 31 December, 2021. Data from the Cancer Registry of Norway. Other hospitals refers to local hospitals which are not specialized in pancreatic cancer related issues and pancreatic surgery.

**Supplementary Table 1.** Baseline characteristics and overall survival for 42 patients receiving only best supportive care. Values are n (%) or median (interquartile range).

|  |  |
| --- | --- |
| Age, years | 78.0 (71-81.3) |
| Sex ratio (Male:Female) | 18:24 |
| Body mass index | 23.0 (20.4-25.4) |
| Charlson comorbidity index |  |
| 0 | 14 (33.3) |
| 1 | 16 (38.1) |
| >1 | 12 (28.6) |
| Performance status (ECOG) |  |
| 0 | 10 (23.8) |
| 1 | 9 (21.4) |
| 2 | 11 (26.2) |
| 3 | 11 (26.2) |
| 4 | 1 (2.4) |
| Biliary drainage | 23 (54.8) |
| CA19-9, kU/L | 646 (109-1505) |
| Tumour location |  |
| Head/uncinate process | 34 (81) |
| Body/tail | 8 (19) |
| Tumour size, mm | 38.5 (30-49.3) |
| Tumour classification |  |
| Borderline resectable | 17 (40.5) |
| Locally advanced | 25 (59.5) |
| Reasons for assigned therapy |  |
| Age | 7 (16.7) |
| Age and performance status | 7 (16.7) |
| Age and comorbidity | 6 (14.3) |
| Comorbidity | 1 (2.4) |
| Performance status | 15 (35.6) |
| Patient decision | 6 (14.3) |
| Overall survival, months (95 % confidence interval) | 3.6 (3.0-5.1) |

ECOG, Eastern Cooperative Oncology Group.

Body mass index, missing data n=8; CA19-9, missing data n=8, non-secretor n=2.

**Supplementary Table 2.** Baseline and treatment characteristics stratified by assigned primary chemotherapy regimen (FOLFIRINOX, gemcitabine/nab-paclitaxel, or gemcitabine). Values are n (%) or median (interquartile range).

|  | **Overall***  **n=178** | **FOLFIRINOX**  **n=103** | **GnP**  **n=48** | **Gemcitabine**  **N=27** | **p-value** |
| --- | --- | --- | --- | --- | --- |
| **Baseline characterstics** |  |  |  |  |  |
| Anatomic tumor classification |  |  |  |  |  |
| Borderline resectable | 91 (51.1) | 52 (50.5) | 26 (54.2) | 13 (48.1) | 0.865 |
| Locally advanced | 87 (48.9) | 51 (49.5) | 22 (45.8) | 14 (51.9) |  |
| Age, years | 69.5 (63-74) | 64 (56-71) | 72.5 (69.5-76) | 77 (72-79.5) | <0.001 |
| Sex ratio (Male:Female) | 98:90 | 59:44 | 24:24 | 12:15 | 0.424 |
| Body mass index | 23.7 (21-26.5) | 24.7 (22.4-27.5) | 23.3 (20.7-25.1) | 21.6 (18.3-24.9) | 0.002 |
| Charlson comorbidity index |  |  |  |  |  |
| 0 | 89 (50) | 57 (55.3) | 20 (41.6) | 12 (44.4) | 0.233 |
| 1 | 58 (32.6) | 33 (32) | 15 (31.3) | 10 (37) |  |
| >1 | 31 (17.4) | 13 (12.7) | 13 (27.1) | 5 (18.6) |  |
| Performance status (ECOG) |  |  |  |  |  |
| 0 | 104 (58.4) | 68 (66) | 24 (50) | 12 (44.4 ) | 0.003 |
| 1 | 61 (34.3) | 33 (32) | 19 (39.6) | 9 (33.3) |  |
| >1 | 13 87.3) | 2 (2) | 5 (10.4) | 6 (22.2) |  |
| Biliary drainage | 97 (54.5) | 54 (52.4) | 29 (60.4) | 14 (51.9) | 0.627 |
| CA19-9 baseline , kU/L | 343 (75-1001) | 252 (74-899) | 432 (101-1406) | 513 (171-1698) | 0.208 |
| Tumour location |  |  |  |  |  |
| Head/uncinate process | 144 (80.9) | 78 (75.7) | 44 (91.7) | 22 (81.5) | 0.068 |
| Body/tail | 34 (19.1) | 25 (24.3) | 4 (8.3) | 5 (18.5) |  |
| Tumour size baseline, mm | 35.5 (29-.44.8) | 35 (29-44) | 34 (28.5-41.5) | 38 (28.5-48) | 0.784 |
| Number of cycles | - | 4 (3-5) | 2 (2-3.8) | 2 (1-3) | NA |
| Chemotherapeutic switch | 29 (16.3) | 24 (23.3) | 3 (6.3) | 2 (7.4) | 0.012 |
| CTCAE Grade 3-5 adverse events | 94 (52.8) | 55 (53.4) | 24 (50) | 15 (55.6) | 0.883 |
| Tumour size post chemotherapy, mm | 33 (25-45) | 32.5 (25-42) | 32.5 (22.5-47.5) | 36 (23-45) | 0.996 |
| CA19-9 post chemotherapy, kU/L | 210 (56-617) | 181 (46-544) | 293 (64-808) | 245 (94-872) | 0.442 |
| CA19-9 dynamics |  |  |  |  |  |
| < 50 % decrease, stable or increase | 107 (64.1) | 65 (67.7) | 29 (64.4) | 13 (61.9) | 0.848 |
| > 50 decrease | 55 (34) | 31 (32.3) | 16 (35.6) | 8 (38.1) |  |
| Normalization | 14 (8.5) | 11 (11) | 3 (6.8) | 0 (0) | 0.232 |
| RECIST response at restaging |  |  |  |  |  |
| Complete/Partial response | 18 (10.3) | 13 (12.7) | 4 (8.3) | 1 (4) | 0.567 |
| Stable disease | 114 (65.1) | 65 (63.7) | 30 (62.5) | 19 (76) |  |
| Progressive disease | 43 (24.6) | 24 (23.6) | 14 (29.2) | 5 (20) |  |

*In addition two borderline resectable patients underwent upfront surgery, and eight patients received other chemotherapy regimens. BSC, best supportive care; CT-CAE, Common, Terminology Criteria for Adverse Events; ECOG, Eastern Cooperative Oncology Group; FOLFIRINOX, 5-fluorouracil with leucovorin, irinotecan, and oxaliplatin; GnP; Gemcitabine nab-paclitaxel; IQR, interquartile range; MDT, multidisciplinary team; NA, not applicable. RECIST; Response Evaluation Criteria In Solid Tumours. Body mass index, missing data n=3; CA19-9 at baseline: missing n=11, hyperbilirubinaemia n=2, non-secretor n=1.

**Supplementary Table 3**. Primary chemotherapy related adverse events grade 3 and 4 observed in 100* of 186 patients. Values are n.

|  |  | **FOLFIRINOX**†  **55 of 103** | | **GnP**  **24 of 48** | | **Gemcitabine**  **15 of 27** | | **FLOX**§  **5 of 6** | |
| --- | --- | --- | --- | --- | --- | --- | --- | --- | --- |
|  |  | Grade 3 | Grade 4 | Grade 3 | Grade 4 | Grade 3 | Grade 4 | Grade 3 | Grade 4 |
| **Overall*** | 145 | 73 | 7 | 28 | 7 | 19 | 5 | 4 | 1 |
|  |  |  |  |  |  |  |  |  |  |
| **Hematologic** | 38 | 15 | 3 | 7 | 2 | 7 | 1 | 2 | 1 |
| Anaemia | 2 |  |  | 1 |  | 1 |  |  |  |
| Neutropenia | 26 | 10 | 2 | 4 |  | 6 | 1 | 2 | 1 |
| Febrile neutropenia | 7 | 5 |  | 1 | 1 |  |  |  |  |
| Thrombocytopenia | 3 |  | 1 | 1 | 1 |  |  |  |  |
|  |  |  |  |  |  |  |  |  |  |
| **Non hematologic** | 106 | 58 | 4 | 21 | 5 | 12 | 4 | 2 | 0 |
| Diarrhoea | 20 | 15 | 1 | 3 | 1 |  |  |  |  |
| Nausea/Vomiting | 8 | 6 |  |  |  | 1 |  | 1 |  |
| Fatigue | 4 | 2 |  | 2 |  |  |  |  |  |
| Anorexia | 2 | 1 |  | 1 |  |  |  |  |  |
| Cholangitis/cholecystitis | 17 | 6 | 3 | 1 | 1 | 4 | 2 |  |  |
| Gallbladder perforation | 2 |  |  |  | 1 |  | 1 |  |  |
| Cholestasis | 2 | 2 |  |  |  |  |  |  |  |
| Elevated liver enzymes | 2 |  |  |  |  | 2 |  |  |  |
| Macules/papules >30% of body | 1 |  |  | 1 |  |  |  |  |  |
| Sensory neuropathy | 3 | 1 |  | 2 |  |  |  |  |  |
| Infections |  |  |  |  |  |  |  |  |  |
| Infection NS | 6 | 3 |  | 2 |  | 1 |  |  |  |
| Infection VAP | 1 | 1 |  |  |  |  |  |  |  |
| Pneumonia | 4 | 2 |  | 2 |  |  |  |  |  |
| Urinary tract infection/ Urosepsis | 3 | 2 |  |  |  | 1 |  |  |  |
| Sepsis | 4 | 2 |  | 1 |  | 1 |  |  |  |
| Liver abscess | 1 | 1 |  |  |  |  |  |  |  |
| Erysipelas | 3 |  |  | 1 |  | 2 |  |  |  |
| Throat infection | 1 | 1 |  |  |  |  |  |  |  |
| Abdominal infection NS | 2 | 1 |  | 1 |  |  |  |  |  |
| Cellulitis | 1 |  |  | 1 |  |  |  |  |  |
| Flu | 1 | 1 |  |  |  |  |  |  |  |
| Abdominal |  |  |  |  |  |  |  |  |  |
| Abdominal pain | 3 | 3 |  |  |  |  |  |  |  |
| Pancreatitis | 1 | 1 |  |  |  |  |  |  |  |
| Oesophagitis | 1 |  |  |  |  |  |  | 1 |  |
| Colitis | 3 | 3 |  |  |  |  |  |  |  |
| Bleeding oesophageal varices | 1 |  |  |  | 1 |  |  |  |  |
| Cardiac pain | 1 | 1 |  |  |  |  |  |  |  |
| Heart failure | 2 |  |  | 1 |  |  | 1 |  |  |
| Kidney failure | 2 | 1 |  | 1 |  |  |  |  |  |
| Hypokalaemia | 1 | 1 |  |  |  |  |  |  |  |
| Hyperglycaemia | 1 | 1 |  |  |  |  |  |  |  |
| Diabetic ketoacidosis | 1 |  |  | 1 |  |  |  |  |  |
| Hypotension | 1 |  |  |  | 1 |  |  |  |  |

*In addition one patient received nab-paclitaxel monotherapy and developed neutropenia grade 3, and one patient received regional chemotherapy without any toxicity.

†One patient died of myocardial infarction after one cycle FOLFIRINOX (grade 5).

§One patient died of Clostridium difficile colitis, diarrhoea and severe dehydration after two cycles FLOX (grade 5).

FLOX, fluorouracil/leucovorin/oxaliplatin; FOLFIRINOX, 5-fluorouracil with leucovorin, irinotecan, and oxaliplatin; GnP, Gemcitabine nab-paclitaxel; NS, not specified.
